# Supplementary material for: Structure of transmembrane prolyl 4-hydroxylase reveals unique organization of EF and dioxygenase domains
Source: J Biol Chem. 2020 Dec 20;296:100197. doi: 10.1074/jbc.RA120.016542 (PMC7948501; doi:10.1074/jbc.RA120.016542)
Supplement: Supplementary file 1 — Figures S1 to S8 [file mmc1.pdf]

Supporting information

Structure of transmembrane prolyl 4-hydroxylase reveals unique organization of EF and dioxygenase domains

Matti Myllykoski[1,2], Aleksi Sutinen[1], M. Kristian Koski[1], Juha P. Kallio[3], Arne Raasakka[3], Johanna Myllyharju[1,2], Rikkert Wierenga[1] and Peppi Koivunen[1,2]*

[1]Biocenter Oulu, Faculty of Biochemistry and Molecular Medicine, [2]Oulu Center for Cell-Matrix Research, University of Oulu, Oulu, Finland. [3]Department of Biomedicine, University of Bergen, Bergen, Norway

Table of content

Figure S1……………………………………………………………………………………2
Figure S2……………………………………………………………………………………3
Figure S3……………………………………………………………………………………4
Figure S4……………………………………………………………………………………5
Figure S5……………………………………………………………………………………6
Figure S6……………………………………………………………………………………7
Figure S7……………………………………………………………………………………8
Figure S8……………………………………………………………………………………9

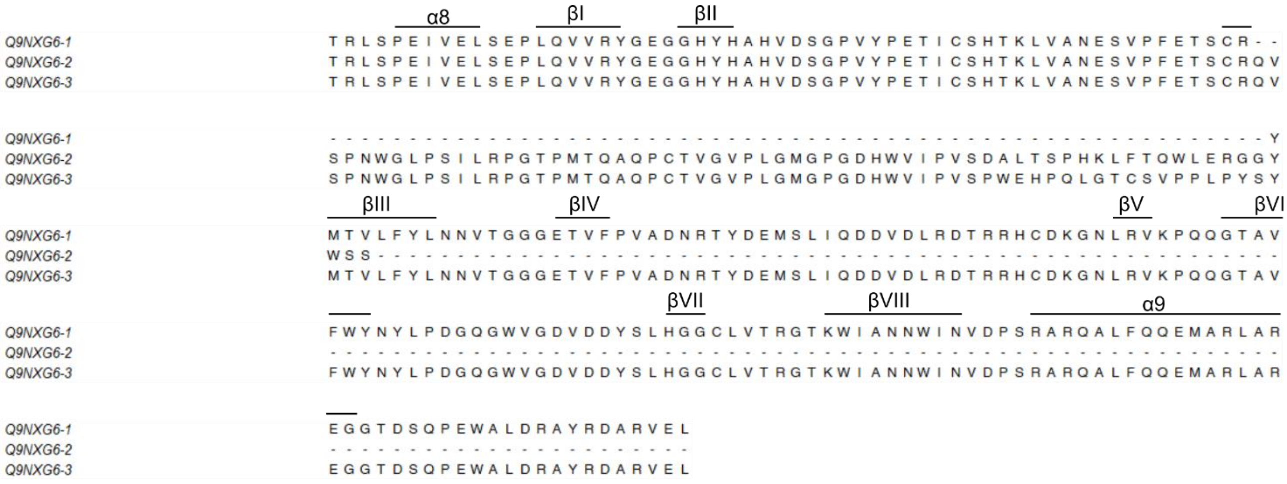

Figure S1. Alignment of the three suggested isoforms of human P4H-TM. The alignment is only shown from residue 301 onwards, because the sequences are identical until the end of exon 6. Q9NXG6-1 refers to the 502-aa P4H-TM crystallized here while Q9NXG6-2 is a 423 aa isoform and Q9NXG6-3 a 563-aa isoform.

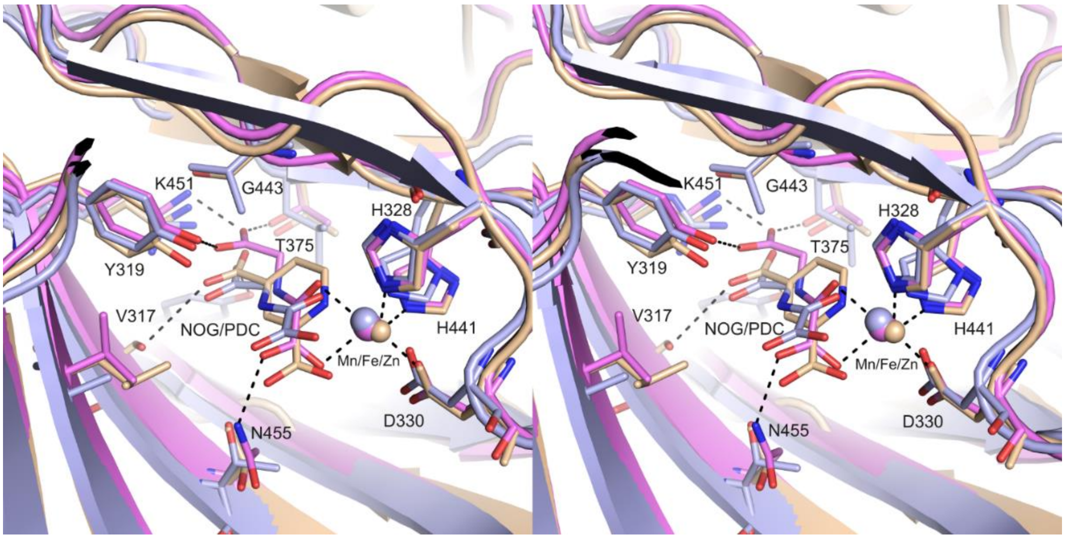

Figure S2. A stereo figure of the metal and co-substrate coordination in P4H-TM and homologs. A stereo figure showing the different metals manganese, iron and zinc (Mn/Fe/Zn) and the 2-oxoglutarate co-substrate analogs N-oxalylglycine and pyrimidine dicarboxylate (NOG/PDC) in the active sites of P4H-TM (violet), *C. reinhardtii* P4H (light brown) and HIF-P4H-2 (light blue). The numbered residues are the residues that interact with the iron and NOG in P4H-TM.

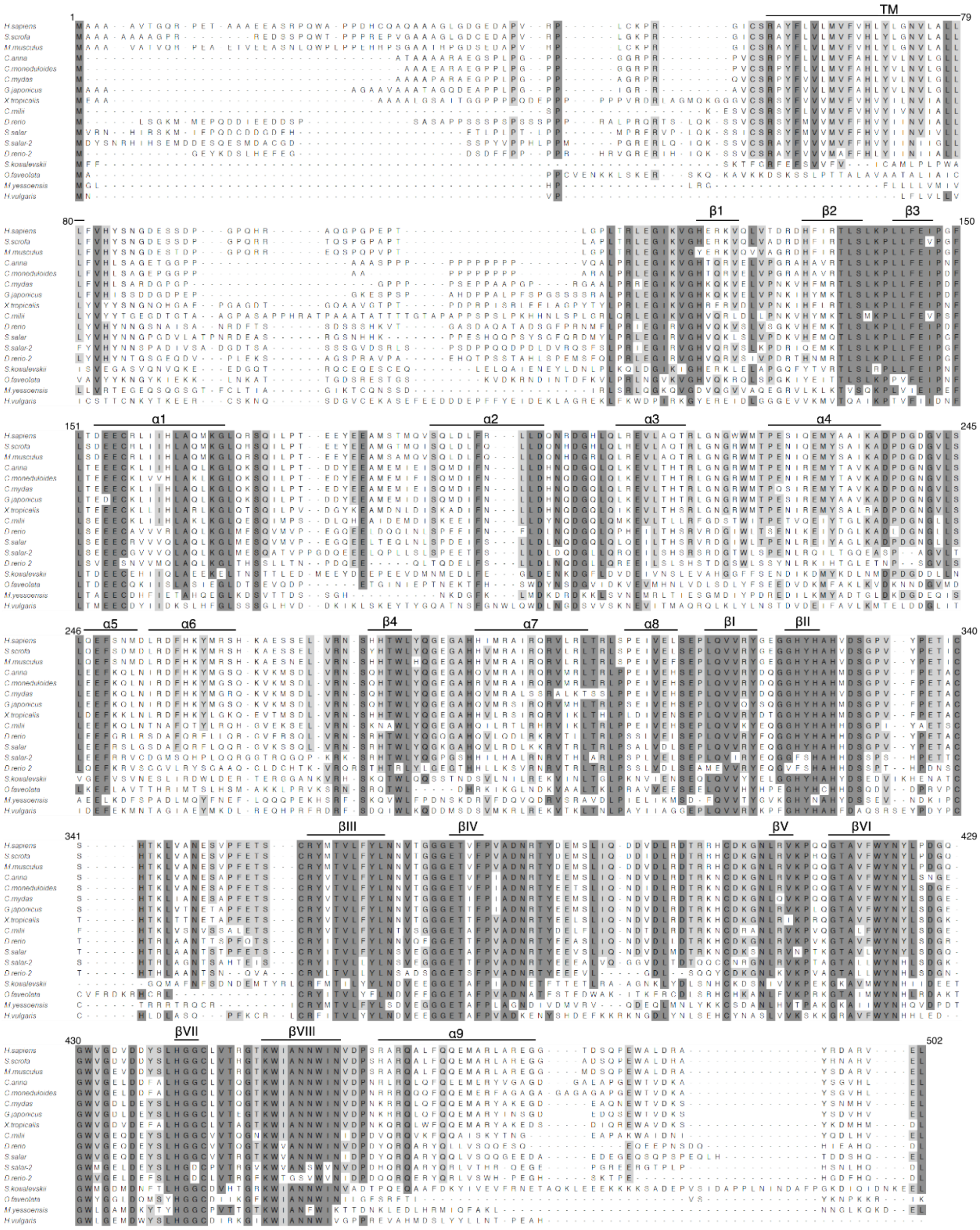

Figure S3. P4H-TM ortholog sequences from different animal species. Some ray-finned fish have two similar but not identical P4H-TM proteins. The zebrafish (*D. rerio*) and salmon (*S. salar*) sequences with lower identity to human P4H-TM are marked with and extension '-2'. P4H-TM sequence was detected also from a few invertebrate species including cnidarian *H. vulgaris*, but it seems to be absent from *e.g.*, the arthropod and nematode phyla.

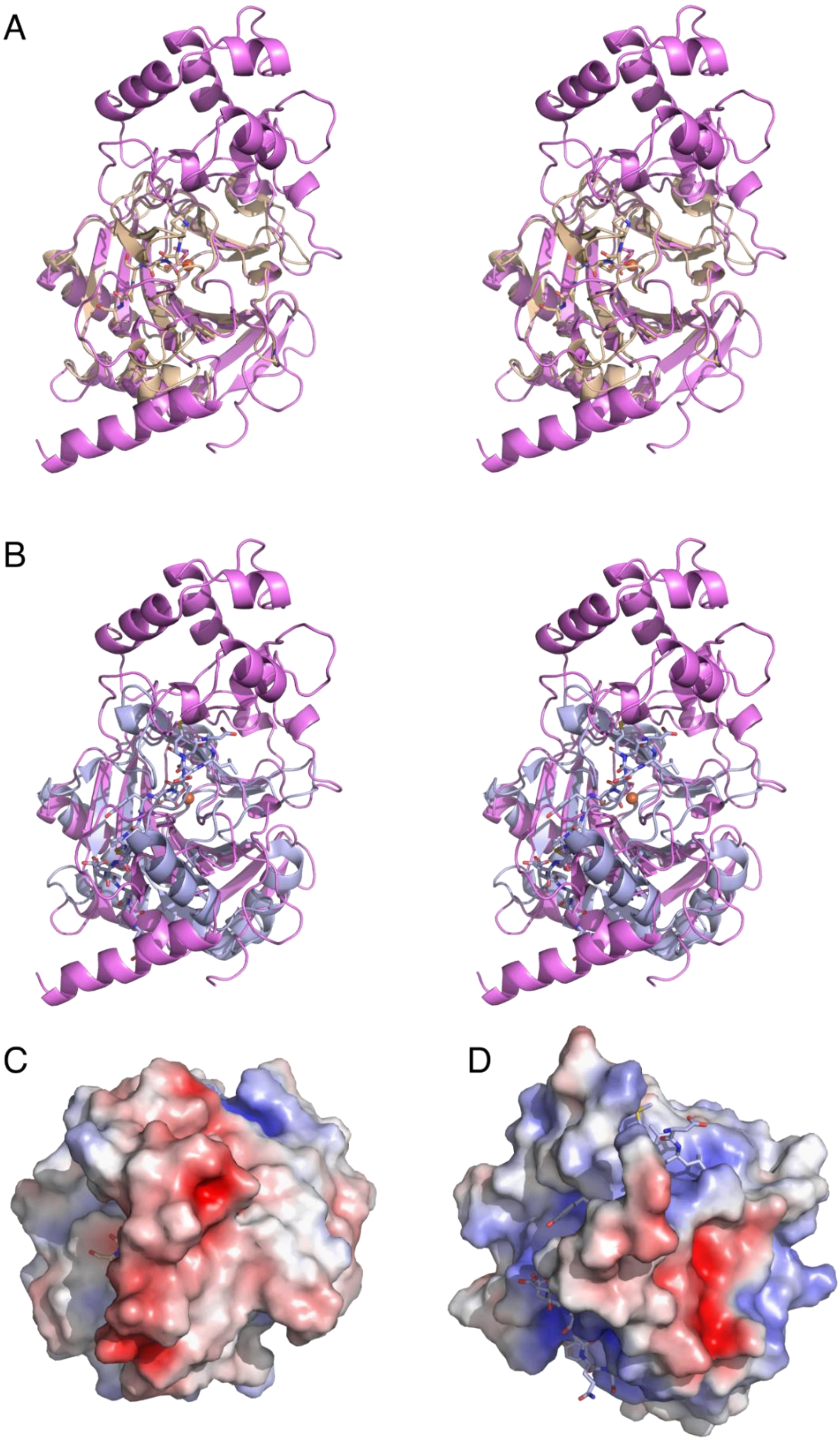

Figure S4. Loop structure and electrostatic surface comparisons of P4H-TM and homologs. The overlaid loop structures of A) P4H-TM (violet) and Cr-P4H (light brown, PDB entry 3gze), and B) P4H-TM and HIF-P4H-2 (light blue, PDB entry 3hqr) in stereo. Electrostatic surfaces and the substrate peptides as a stick model of C) Cr-P4H and D) HIF-P4H-2. The orientation of the proteins is the same in this figure and in Figs. 6A and 6B in the main text.

A

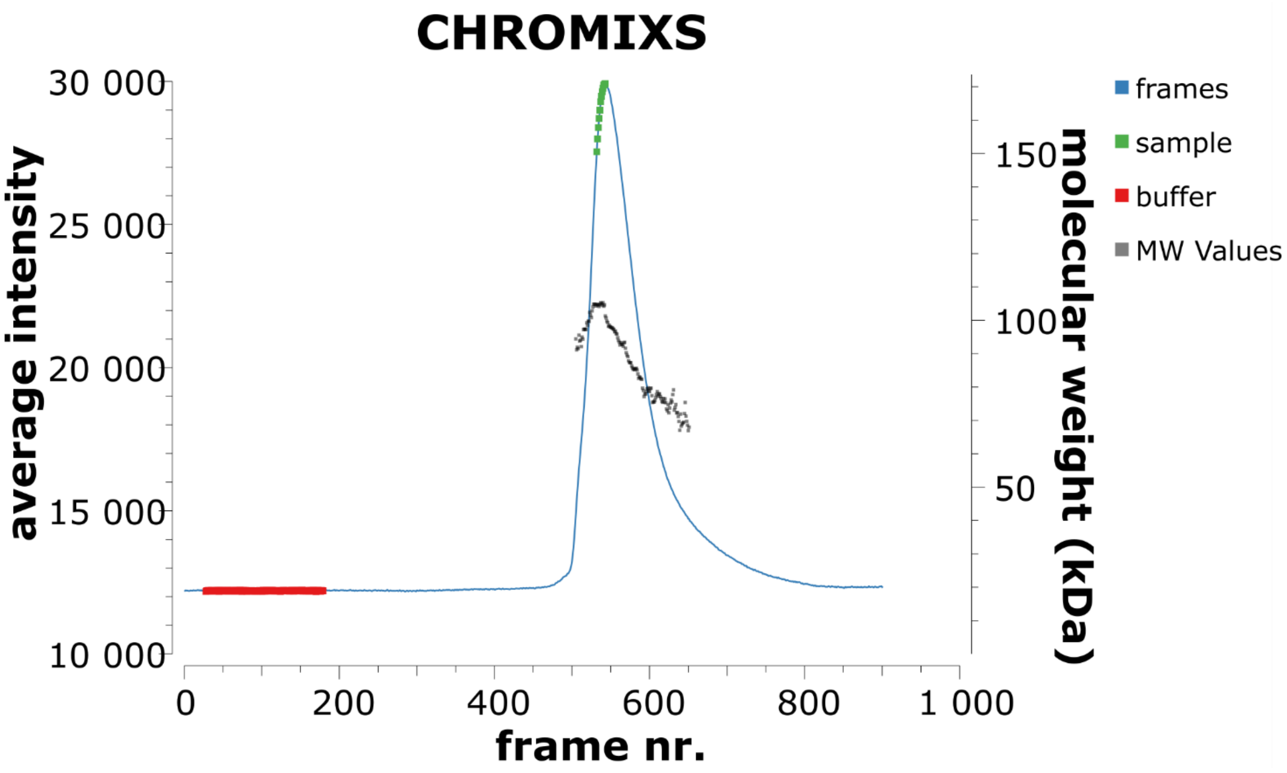

B

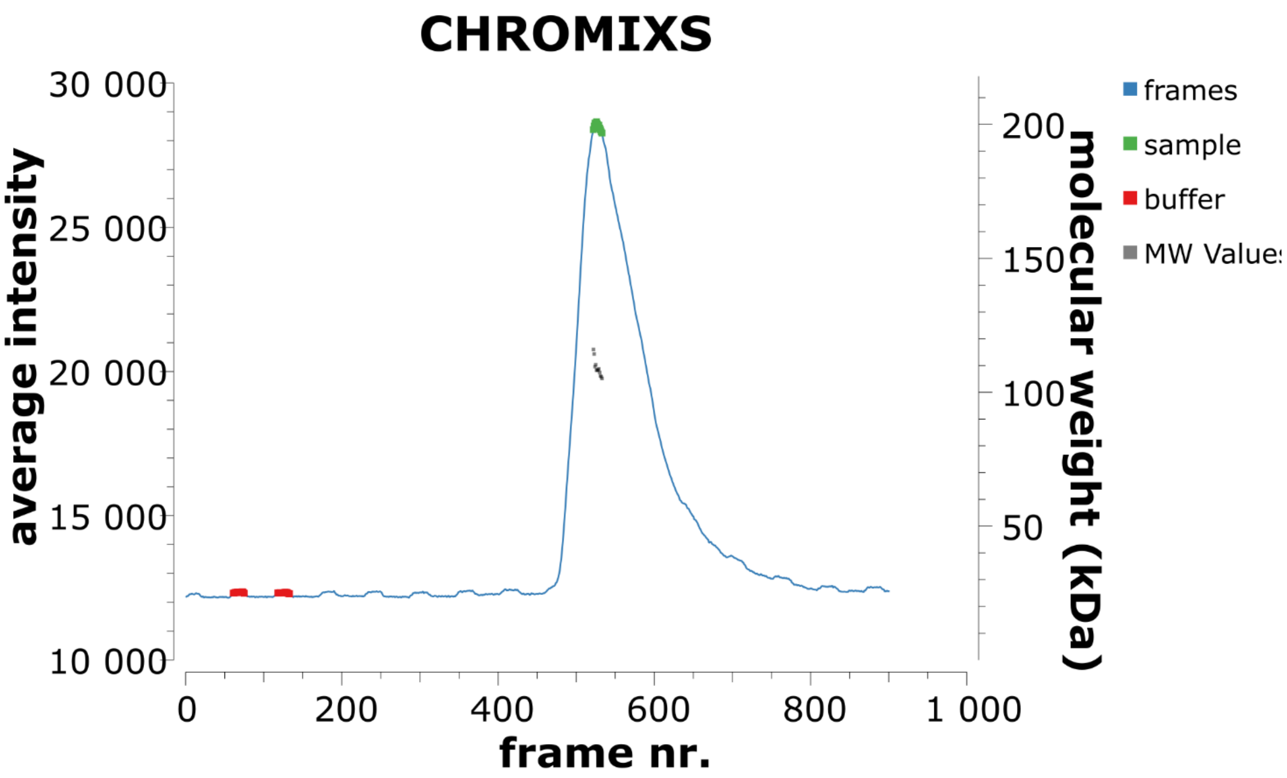

Figure S5. SEC-SAXS chromatography elution curves for P4H-TM without and with calcium in running buffer. A) without calcium, B) with calcium.

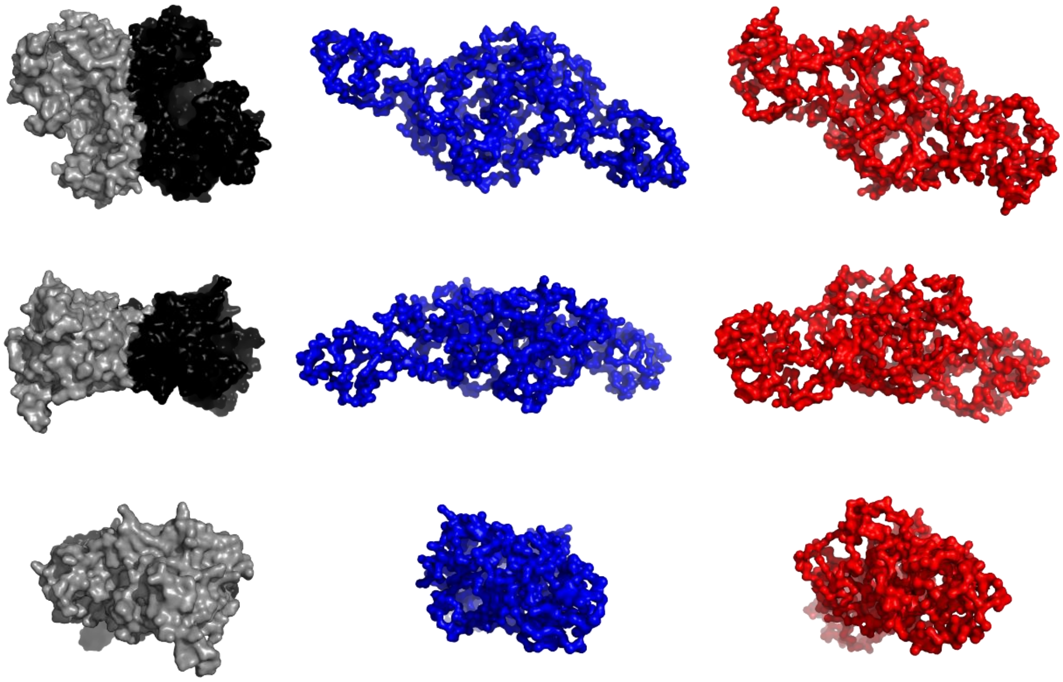

Figure S6. P4H-TM crystal structure surface and SAXS model envelopes in different orientations. In black and grey are the two P4H-TM molecules in the asymmetric unit of the crystal structure. The blue shape is the P4H-TM SAXS model without calcium and red model is the P4H-TM SAXS model with calcium. SAXS models were generated with GASBOR.

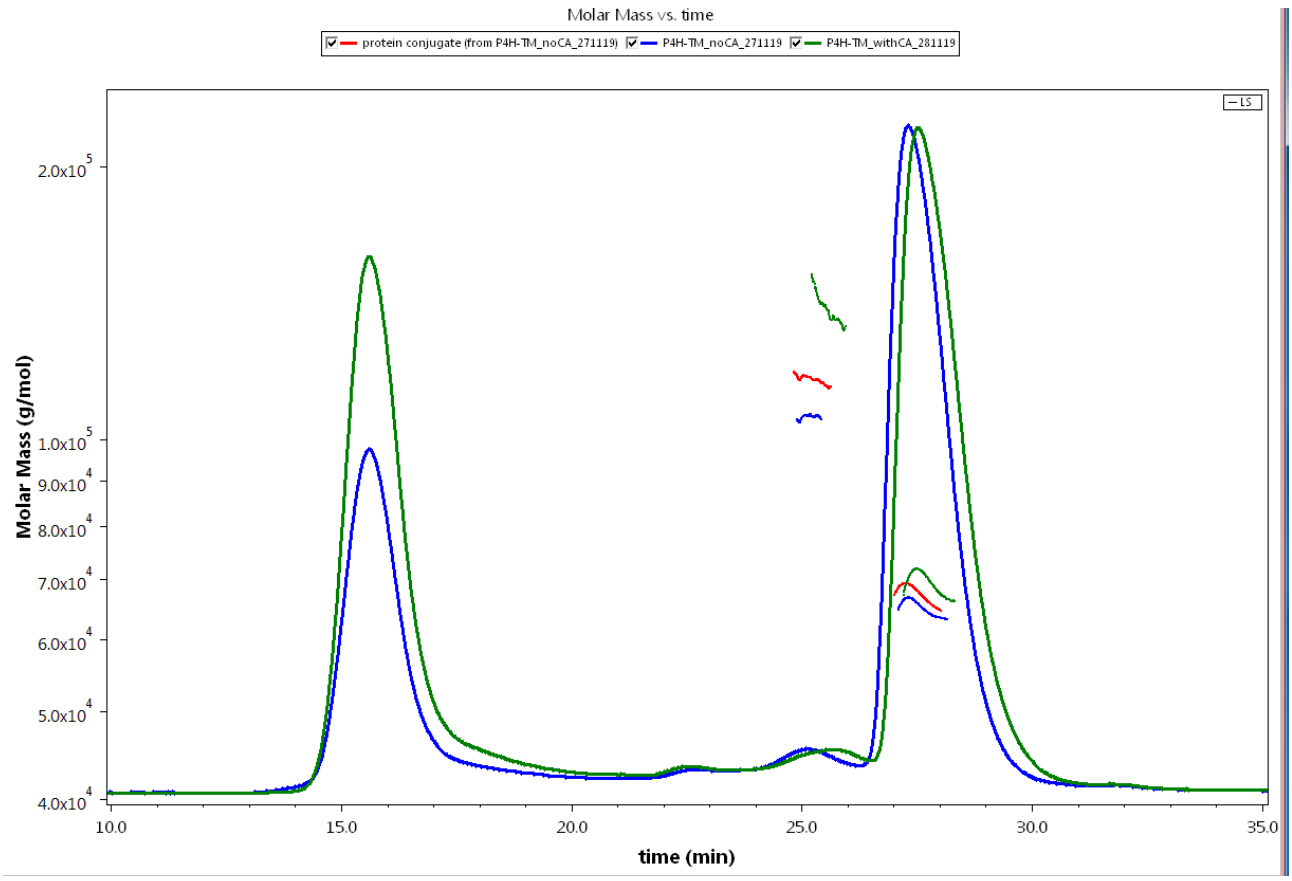

Figure S7. Online MALS/SEC analysis of P4H-TM. P4H-TM with Ca^{2+} (green) and without Ca^{2+} (blue) using the Shimadzu HPLC equipped with a Superdex 200 Increase 10/300 GL (GE Healthcare) and a Wyatt miniDAWN for recording the MALS signal. The plot of molar mass (horizontal red line) versus elution time is shown for both runs and for the without calcium run analyzed with the protein conjugate method (red). The MALS signals show that the major form of P4H-TM that eluted at 28 min corresponds to a monomer protein in both cases with the calculated molecular weight of 65 kDa without Ca^{2+} (blue curve) and 69 kDa in the presence of Ca^{2+} (green curve). The chromatograms also show an aggregate peak at 15-16 ml and a very minor peak around 25 ml, which could correspond the dimeric (or higher oligomer) form of P4H-TM (molecular weights of 106 kDa and 140 kDa).

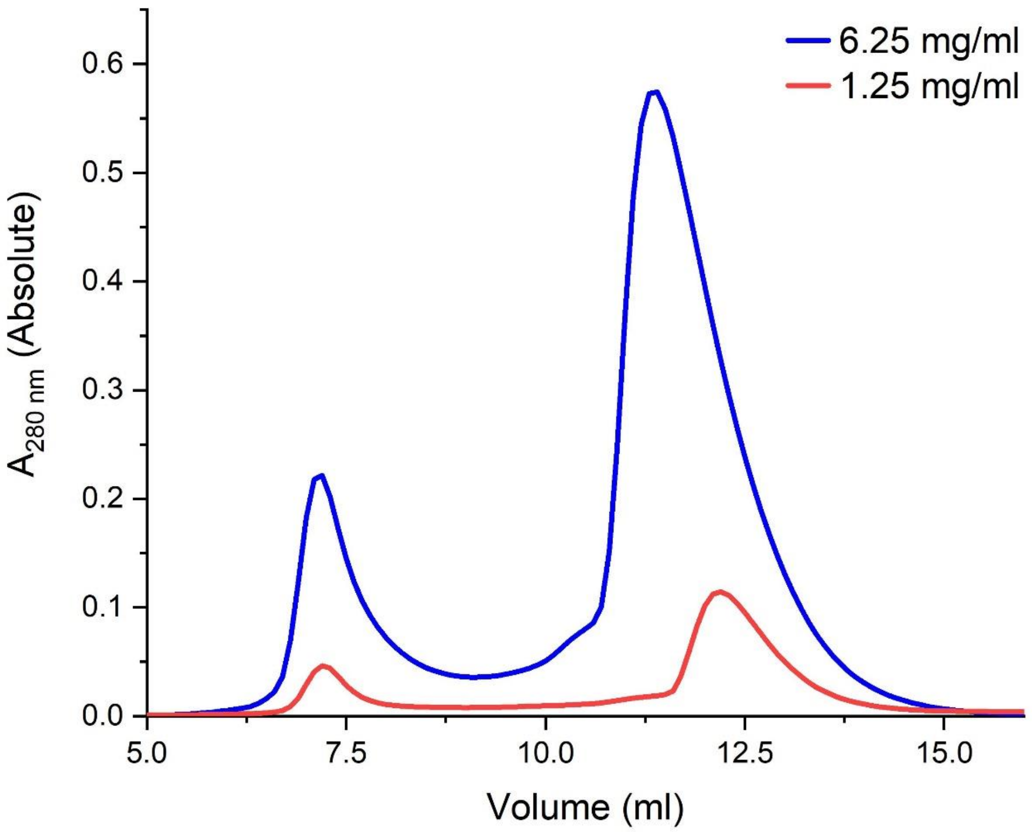

Figure S8. **SEC analyses of P4H-TM at two different concentrations.** Two overlaid chromatograms of P4H-TM at different concentrations injected to the Superdex 200 10/300 column equilibrated with the SEC buffer. Samples were aliquots from the same affinity column elution, one aliquot being injected to the column without further concentration and the other one after 5-fold concentration. Although both samples produced a single peak after the aggregation peak at 7 ml, the peak volume shifted to 11.3 ml in the more concentrated sample compared to 12.2 ml in the non-concentrated sample.
